# Transformer (Tra2β): master regulator of myosin phosphatase alternative splicing and smooth muscle responses to NO/cGMP signaling

**DOI:** 10.1186/1471-2210-11-S1-P24

**Published:** 2011-08-01

**Authors:** Steven A Fisher, Ylva Mende, Brunhilde Wirth, Kang Fu

**Affiliations:** 1 Department of Medicine, Case Western Reserve University, Cleveland, Ohio 44106, USA; 2 Institute of Human Genetics, University of Cologne, Cologne, Germany

## Background

Nitric oxide signaling through the cGMP kinase (cGK1α) activates Myosin Phosphatase (MP) leading to calcium de-sensitization of force production. Leucine zipper (LZ) motifs present in the C-terminus of MYPT1 and N-terminus of cGK1α are thought to be essential for the hetero-dimerization of cGK1 and MYPT1 and cGK1 activation of MP [1]. An isoform of MYPT1 that lacks the C-terminal LZ motif is generated by the alternative splicing of a 31 nt 3’ exon (E23). We have shown that the expression of these MYPT1 isoforms is tissue-specific, developmentally regulated and modulates in disease [2]. MYPT1 E23-/LZ+ isoform is expressed in tonic smooth muscle of the large arteries and veins while MYPT1 E23+/LZ- isoform is expressed in the phasic smooth muscle of the intestines, portal vein and small resistance arteries. There is a good though incompletely characterized correlation between the expression of MYPT1 isoforms and sensitivity to NO/cGMP-mediated relaxation, making this alternative splicing event an excellent model for the study of smooth muscle phenotypic diversity in relation to vascular function.

## Results

In a previous study we identified Transformer (Tra2β), a master regulator of splicing and sex determination in *Drosophila*, as a candidate tissue-specific regulator of MYPT1 E23 splicing [3]*.* We now examine this question in the mouse in order to take advantage of a conditional *Sfrs10* (Tra2β) knockout model [1]. As in the rat 1) Tra2β is expressed at higher levels in phasic smooth muscle of small arteries and intestines, where E23 is spliced as compared to tonic smooth muscle of large vessels, where E23 is skipped. 2) Tra2β is developmentally up-regulated as smooth muscle acquires a phasic phenotype/E23 splicing. To test the role of Tra2β *in vivo*, SM22Cre and Tra2β flox mice were crossed for conditional inactivation of Tra2β in smooth muscle. The homozygous (Cre+//f/f) mice are non-viable. Heterozygous mice (Cre+//f/+) have significantly decreased levels of MYPT1 E23 inclusion in many of the phasic smooth muscle tissues.

## Conclusion

Tra2β is necessary for tissue-specific splicing of MYPT1 E23. Tra2β may function as a master regulator of smooth muscle phenotype and responses to NO/cGMP signaling.

This work was supported by NIH grant RO1 HL-66171 to SAF and CMMC grant D7 and DFG grant Wi945/13-1 to BW.
